# Torque Teno Virus (TTV) in Renal Transplant Recipients: Species Diversity and Variability

**DOI:** 10.3390/v16030432

**Published:** 2024-03-11

**Authors:** Noelia Soledad Reyes, Pietro Giorgio Spezia, Raquel Jara, Fabio Filippini, Natalia Boccia, Gonzalo García, Eliana Hermida, Fernando Adrian Poletta, Mauro Pistello, Gustavo Laham, Fabrizio Maggi, Marcela Echavarria

**Affiliations:** 1 Virology Unit, Centro de Educación Médica e Investigaciones Clínicas (CEMIC) University Hospital, Consejo Nacional de Investigaciones Científicas y Técnicas (CONICET), Av. Galván 4102, Buenos Aires C1631FWO, Argentina; jararaquel.93@gmail.com (R.J.); elu.luz.eh@gmail.com (E.H.); mechavarria@cemic.edu.ar (M.E.); 2 Laboratory of Virology, National Institute for Infectious Diseases Lazzaro Spallanzani—IRCCS, 00149 Rome, Italy; fabrizio.maggi@inmi.it; 3 Department of Translational Research, University of Pisa, 56127 Pisa, Italy; f.filippini1996@gmail.com (F.F.); mauro.pistello@unipi.it (M.P.); 4 Department of Nephrology, Centro de Educación Médica e Investigaciones Clínicas (CEMIC) University Hospital, Buenos Aires C1631FWO, Argentina; natiboccia@gmail.com (N.B.); garcia.gonzalo.90@gmail.com (G.G.); gustilaham@gmail.com (G.L.); 5 Genetic Epidemiology Laboratory, Centro de Educación Médica e Investigaciones Clínicas (CEMIC) University Hospital, Consejo Nacional de Investigaciones Científicas y Técnicas (CONICET), Buenos Aires C1631FWO, Argentina; fpoletta@eclamc.org

**Keywords:** Torque Teno Virus, TTV species, renal transplantation, anelloviruses

## Abstract

Torque Teno Virus (TTV) is a nonpathogenic and ubiquitous ssDNA virus, a member of the *Anelloviridae* family. TTV has been postulated as a biomarker in transplant patients. This study aimed to determine the TTV species diversity and variability in renal transplant recipients and to associate species diversity with the corresponding TTV viral load. From 27 recipients, 30 plasma samples were selected. Viral load was determined using two real-time PCR assays, followed by RCA-NGS and ORF1 phylogenetic analysis. The TTV diversity was determined in all samples. Variability was determined in three patients with two sequential samples (pre- and post-transplantation). Most of the samples presented multiple TTV species, up to 15 different species were detected. In the pre-transplant samples (*n* = 12), the most prevalent species were TTV3 (75%) and TTV13 (75%), and the median number of species per sample was 5 (IQR: 4–7.5). TTV3 was also the most prevalent (56%) in the post-transplant samples (*n* = 18), and the median number of species was 2 (IQR: 1.8–5.5). No significant correlation between the number of species and viral load was found. The number and type of TTV species showed total variability over time. We report high TTV species diversity in Argentinian recipients, especially in pre-transplant period, with total intra-host variability. However, we found no significant correlation between this high diversity and TTV viral load.

## 1. Introduction

Torque Teno Virus (TTV) is a non-enveloped virus, with a small, single-stranded, negative-sense DNA genome. This virus is nonpathogenic in humans and highly ubiquitous [1]. TTV has been postulated as a surrogate marker of immunosuppression in transplant patients, especially among solid organ recipients [2].

TTV genome is approximately 3.8 Kb in length, organized into a coding region sequence (CDS) of around 2.6 Kb and a highly conserved non-coding region (UTR) of approximately 1.2 kb [1,3,4]. The CDS presents at least four partially overlapping open reading frames (ORF1–4) [5,6]. Like most circular DNA viruses, TTV DNA replication can be carried out by a rolling circle mechanism [7].

The TT designation of this virus was in honor of a Japanese patient with transfusion-acquired non-AG hepatitis, from whom the virus was first isolated in 1997 [8]. TTV is a member of the highly diverse *Anelloviridae* family. Three members of this family can infect humans: TTV (genus Alphatorquevirus), Torque Teno Mini Virus (TTMV, genus Betatorquevirus) and Torque Teno Midi Virus (TTMDV, genus Gammatorquevirus) [9,10].

According to the previous International Committee on Taxonomy of Viruses (ICTV) guidelines, the 29 TTV species described were divided into five genogroups: genogroup I (TTV 1–5), genogroup II (TTV 6–8), genogroup III (TTV 13–24), genogroup IV (TTV 25–29) and genogroup V (TTV 9–12). In the latest ICTV update, TTV species were re-categorized into 22 species, based on a sequence identity threshold of 69% for the ORF1 coding region [11].

TTV can be detected in peripheral blood regardless of age, sex or socioeconomic level, with 90% prevalence in healthy individuals. TTV transmission occurs mainly through the fecal–oral and respiratory routes. Transmission may also occur in utero, through the umbilical cord or amniotic fluid [12,13,14,15].

Several studies on blood donors demonstrated that TTV prevalence is linearly correlated with age. In a Romanian study including 701 blood donors with a median age of 38 years old, the TTV prevalence was 58%. In the largest cohort study published to date, including 1017 Italian blood donors with a median age of 44 years old, the detected TTV prevalence was 65%. A higher TTV prevalence (76%) was detected in a cohort of 313 Austrian blood donors with a median age of 53 years [16,17,18].

Despite being a non-pathogenic virus, currently not associated with any known pathologies, TTV appears to establish persistent infections in its host. Furthermore, it is postulated that TTV inhibits part of the host immune system [5,19].

TTV ORF2 encodes a protein (~200 aa) with a tyrosine phosphatase motif, capable of interfering with the translocation of NF-κB. NF-κB is a well-characterized signal transcription factor, crucial for lymphocyte development, inflammation and cell growth, among other processes. Therefore, by inhibiting the expression of these pro-inflammatory genes, TTV can evade the host’s immune system [20,21].

Another mechanism employed by TTV to enhance evasion and facilitate persistence in the host is the expression of microRNAs (miRNA, small, non-coding, 22-nucleotide-long RNAs). Potential miRNA sequences in the TTV genome have been discovered using computational methods (TTV miRNA t1a of genogroup 1, TTV miRNA t3b of genogroup 3 and TTV miRNA tth8 of genogroup 5). This miRNA-mediated immune evasion may contribute to the ubiquity and persistence of TTV in the population. The role and importance of TTV miRNA expression have not yet been fully studied [22].

Multiple TTV species can simultaneously be detected in humans, which may be acquired at a single time or sequentially over time, resulting in a mixture of TTV species and even different members of the Anelloviridae family. Only a few studies have been conducted in Latin America, reporting the most prevalent TTV species were from genogroup III, followed by species from genogroup I. This was also observed in Europe in healthy populations [23,24,25].

Advances in next-generation sequencing (NGS) have deepened our knowledge on the microbial world. The combination of molecular biology technics like rolling circle amplification (RCA) with NGS on different human samples (both fluid and tissues) demonstrated that anelloviruses are one of the major components of the human virome, with TTV being one of the most prevalent viruses detected [24,26,27].

The aims of this study were to determine the TTV species diversity and variability in renal transplant patients over time and to associate TTV species diversity with the corresponding TTV viral load.

## 2. Materials and Methods

Plasma samples from adult recipients (>18 years old) who underwent renal transplantation between November 2018 and April 2021 at CEMIC University Hospital (Buenos Aires, Argentina) were prospectively collected and evaluated for TTV viral load before and during the first year after transplantation.

The inclusion criteria included renal transplant patients who received thymoglobulin or basiliximab for induction therapy, followed by an immunosuppression maintenance scheme with tacrolimus, mycophenolic acid and steroids.

The exclusion criteria included renal transplant patients with primary renal graft failure or changes in the immunosuppression maintenance scheme. Patients who changed their ambulatory care center before the third month post-transplantation were also excluded.

Diversity was defined as the presence of more than one TTV species in the same sample, and variability was defined as the presence of different species (variation) over time within the same host.

To rule out graft rejection, all patients were evaluated using protocol renal biopsies between 3–6 months and one year after transplantation. Additional renal biopsies were performed in case the patient presented renal dysfunction or to confirm BK nephropathy. Cellular and humoral graft rejections were documented and scored according to the BANFF classification (2019 update). Borderline changes were also included as a graft rejection event since all cases of allograft rejection (cellular, humoral and borderline changes) led to clinical intervention with antirejection treatment.

All recipients were regularly screened for Cytomegalovirus (CMV), both after the completion of routine valganciclovir prophylaxis or if the patient, despite prophylaxis, had symptoms consistent with CMV infection. BK polyomaviruses (BKPyV) were tested by PCR every 2 months for the first six months after renal transplantation and every 3 months thereafter.

### 2.1. Plasma Samples

A total of 30 samples from 27 patients were selected for evaluating the TTV species diversity using next-generation sequencing (NGS).

The selection criteria were a TTV viral load higher than 3 Log_10_ copies/mL or viral load discrepancy between two PCR assays. Twelve were pre-transplant samples and eighteen were post-transplant samples. Of these 18 samples, 9 showed a discrepant result between the two detection methods used.

To evaluate the TTV species variability, 3 patients were evaluated before and after transplantation.

### 2.2. TTV Detection and Quantification

TTV was detected and quantified using two real-time PCR assays:

TTV Home Brew: A home brew PCR method was performed, using primers from a highly conserved UTR region, as previously reported [28]. Positive and negative controls were included in each run. Quantification was performed using a quantified plasmid.

Commercial PCR: The TTV R-GENE^®^ kit (bioMérieux, Marcy-l’Etoile, France). TTV quantification was performed according to the manufacturer’s instructions. The sensitivity control, negative control, positive control and four quantification standards were included in each run. The reported limit of detection is 2.4 Log_10_ copies/mL.

TTV R-GENE^®^ PCR can occasionally detect high levels of TTMV and TTMDV, other members of the *Anelloviridae* family.

### 2.3. TTV Genotyping

A whole-genome, amplicon-based, next-generation sequencing (NGS) technique was used to detect individual TTV species, as previously described [29].

Briefly, rolling circle amplification (RCA) technology was used to enrich the circular DNA of the anelloviruses. The RCA reaction mixture contained a DNA sample of less than 10 ng, 25 µM of exonuclease-resistant random primers (Thermo Fisher Scientific, Carlsbad, CA, USA), 4 mM of deoxynucleotides (dNTPs) (Solis BioDyne, Tartu, Estonia) and 10 U of φ29 DNA polymerase (Thermo Fisher Scientific, Carlsbad, CA, USA). Amplification was carried out at 30 °C for 18 h, followed by inactivation of the φ29 DNA polymerase at 65 °C for 10 min. The resulting product was quantified using a NanoDrop Lite instrument (Thermo Fisher Scientific) and diluted for use as a template in the universal anellovirus inverse (UAvI) PCR.

The UAvI PCR was then performed in order to obtain a full-genome amplicon. The reaction mixture of 50 μL contained 1× PrimeSTAR^®^ GXL Buffer (Takara, Otsu, Japan), a 200 μM dNTP mixture (Takara, Otsu, Japan), 0.3 μM forward primer, 0.3 μM reverse primer, 1.25 U of PrimeSTAR^®^ GXL DNA polymerase (Takara, Otsu, Japan) and 500 to 1000 ng of template. The primers used were 206INV_TTVFor and 205INV_TTVRev. The PCR was carried out for 35 cycles. The PCR product was purified and quantified. Libraries were generated using the Illumina DNA Prep kit, with an initial DNA input of 150 ng. The pool of libraries was quantified using Qubit, and the sizes of the fragments were estimated using the Bioanalyzer. The denatured libraries were then sequenced using an Illumina MiSeq V3 cartridge (600 cycles) with a paired-end 2 × 250 bp and 2 × 150 bp layout.

In the bioinformatic analysis, a pipeline that integrated both reference-based and de novo assembly approaches was used. Firstly, low-quality reads and adapters were trimmed using the fastp software v0.220. Human-origin reads were subsequently removed with the use of the Kraken 2 softwarev2.1.2, using a human genome database (Accession: GRCh38.p13).

The remaining reads were aligned against a comprehensive anelloviruses database. Mapped reads were used to reconstruct complete (or nearly complete) genomic consensus sequences. Non-human reads were then aligned using the BWA-MEM software v.0.7.17 to cross-validate the viral assembly. The obtained ORF1 gene sequences using the orfipy algorithm were then subjected to visual inspection for quality assurance.

### 2.4. Phylogenetic Analysis

The ORF1 phylogenetic analysis was performed using the sequences obtained from the selected plasma samples plus reference sequences (*n* = 190) from GenBank. These reference sequences were manually edited using BioEdit v7.0.5.3 [30] to be approximately as long as ORF1. All sequences were then aligned using ClustalW v1.81 [31].

The Maximum Likelihood method was used with GTR + F + I + G4 as the model of nucleotide substitution. The phylogenetic tree was made using the IQ-TREE software v.1.6.12, supported by UltraFast Boostrap and SH-aLRT branch testing (1000 replicates) [32,33].

### 2.5. Statistical Analysis

Continuous variables were expressed as means and standard deviation or medians and interquartile ranges (IQRs, 25th–75th percentile) according to their distribution. Categorical variables were expressed as frequencies and percentages. The Mann–Whitney method was used to compare the number of TTV species with the sample period (pre- and post-transplantation). The correlations between the number of TTV species and TTV viral load, risk of infection or risk of graft rejection were calculated using Pearson’s correlation coefficient (r). A value of *p* < 0.05 was considered statistically significant. The data were analyzed using the GraphPad Prism software (version 5; GraphPad software, La Jolla, CA, USA).

This study was approved by the CEMIC Ethics Committee (qualified by the Department of Health and Human Services, Buenos Aires, Argentina, HHS no. 124).

## 3. Results

A total of 107 renal transplant patients were enrolled between November 2018 and April 2021 at CEMIC University Hospital for an ongoing prospective study. Of them, 27 patients were selected, and 30 plasma samples were successfully typed by RCA-NGS sequencing.

The patients’ mean age was 49.5 ± 13.9 years old, and 14/27 (51.8%) were female. Most of them received a graft from a deceased donor (74.1%), and 96.3% received thymoglobulin as immunosuppression induction. All the patients were on steroids, tacrolimus and mycophenolic acid for maintenance immunosuppression (Table 1). All the transplant patients were monitored for HIV, HBV and HCV. None of the patients in our cohort were positive for these viruses. No patient lost the graft up to the first year after transplantation.

**Figure 1:**
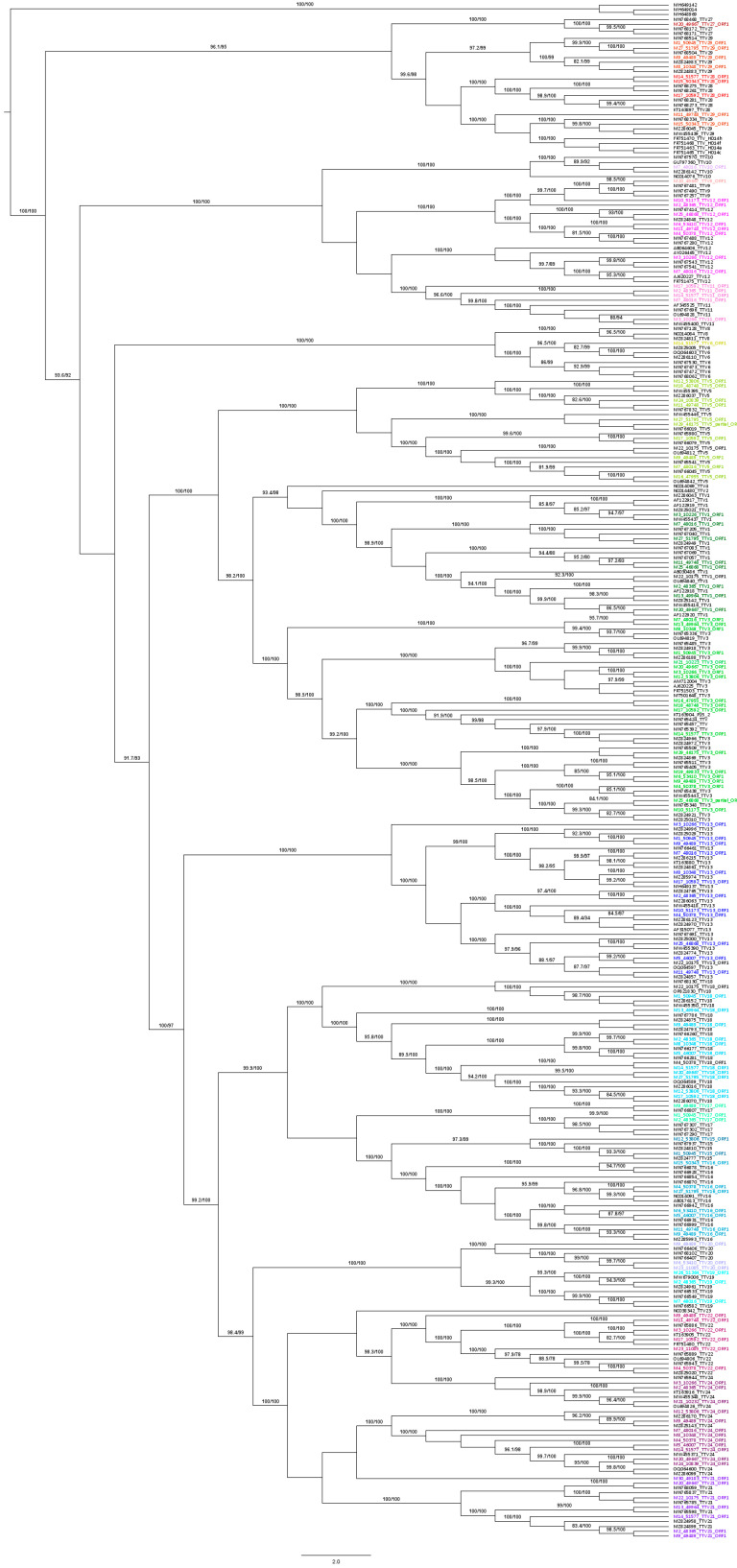
Phylogenetic tree of the TTV species detected in 30 samples from Argentine renal transplant recipients. Reference sequences (*n* = 190) from GenBank. Maximum Likelihood method was used with GTR + F + I + G4 as the model of nucleotide substitution. Phylogenetic tree was made using IQ-TREE software v1.6.12, supported by UltraFast Boostrap and SH-aLRT branch testing (1000 replicates). TTV species detected in samples from our study are colored.

A total of 15 TTV species were detected in the 30 plasma samples (Figure 1).

### 3.1. TTV Diversity

The most prevalent species were TTV 3 (63.3%) and TTV 24 (53.3%), followed by TTV 9 (43.3%) and TTV 13 (43.3%) (Table 2).

The median number of TTV species per patient was 4 (IQR: 2–6.3). Up to 10 TTV species were detected in a single patient.

The difference in the total number of TTV species between the pre- and post-transplant periods was statistically significant (*p *= 0.021).

In the pre-transplant period (*n* = 12), the most prevalent species were TTV 3 (75%) and TTV 13 (75%), followed by TTV 18 (58.3%) and TTV 24 (58.3%). The median number of TTV species per sample was 5 (IQR: 4–7.5). In the samples from patients with a living donor, 3/12 (25%), the median number of TTV species was 4 (IQR: 4–5). In the samples from patients with a cadaveric donor, 9/12 (75%), the median number of TTV species was 6 (IQR: 4.5–8) (Table 3, Table S1).

In the post-transplant period (*n* = 18), the most prevalent species was TTV 3 (55.6%), followed by TTV 24 (44.4%) and TTV 5 (38.9%). The median number of TTV species per sample was 2 (IQR: 1.8–5.5). Particularly, in the period of greatest immunosuppression, at month 3 (*n *= 7), the median number of TTV species per sample was 3.5 (IQR: 2–7) (Table 3).

Different variants of the same species were detected in five samples, particularly species that were grouped in the latest taxonomic update. Specifically, two different variants of TTV 9 (previously named TTV 11 and TTV 12) were detected in three pre-transplant samples. In one of these samples, two variants of TTV 24 (formerly TTV 22 and TTV 24) were also detected. A different pre-transplant sample also had two variants of TTV 24 (TTV 22 and TTV 24). Finally, two variants of TTV 29 (formerly TTV 28 and TTV 29) were detected in a post-transplant sample (Table 3).

#### 3.1.1. TTV Species Diversity and Viral Load

The TTV median viral load in pre-transplant samples was 4.5 Log_10_ copies/mL (IQR: 4.2–5.3) and 4.1 Log_10_ copies/mL (IQR: 3.9–4.8) using the Home Brew PCR and R-GENE^®^ PCR, respectively. At month 3, the TTV median viral load was 8.4 Log_10_ copies/mL (IQR: 8.2–8.5) and 7.4 Log_10_ copies/mL (IQR: 6.9–7.7) using the Home Brew PCR and R-GENE^®^ PCR, respectively (Table 3).

No associations between the number of TTV species and the TTV viral load pre- and post-transplantation were found using either assay.

**Figure 2:**
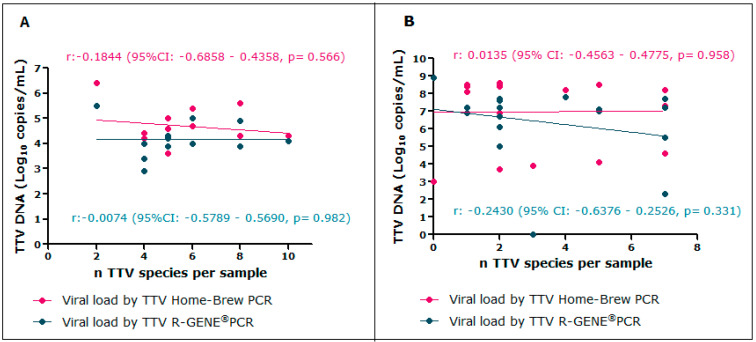
Association between the number of TTV species and TTV viral load. (**A**) Pearson’s correlation between the number of TTV species and TTV viral load pre-transplantation. (**B**) Pearson’s correlation between the number of TTV species and TTV viral load post-transplantation.

In the pre-transplant samples, Pearson’s correlation coefficients were r = −0.1844 (*p *= 0.566) and r = −0.0074 (*p *= 0.982) using the Home Brew PCR and R-GENE^®^ PCR, respectively (Figure 2A). In the post-transplant samples, the coefficients were r = 0.0135 (*p* = 0.958) and r = −0.2430 (*p *= 0.331) using the Home Brew PCR and R-GENE^®^ PCR, respectively (Figure 2B).

#### 3.1.2. TTV Species Diversity and Patient Characteristics

No associations between the number of TTV species and patient age or time on dialysis were found.

Pearson’s correlation coefficients were r = 0.062 (95% CI: −0.3051–0.4131, *p* = 0.745) between the number of TTV species and patient age (Figure S1) and r = 0.1637 (95% CI: −0.2089–0.4949, *p* = 0.387) between the number of TTV species and time on dialysis (Figure S2).

### 3.2. TTV Variability

The variability testing performed in three patients with pre- and post-transplant samples showed a variation in the number of TTV species. Specifically, the total number of TTV species went from 4 species to 1; from 5 species to 0 and from 2 species to 1 in each patient (Table 3).

Accordantly, total variability was also observed in the species detected throughout time. The post-transplant samples showed a different species in two patients and a TTV species that could not be sequenced in one patient.

### 3.3. TTV Species and Viral Infection or Graft Rejection

Among 27 renal transplant recipients, 4 had viral infection (2 CMV cases and 2 BKPyV cases), 3 had graft rejection, 2 had graft rejection and viral infection (CMV and BKPyV) and 18 had neither (Table 3).

No specific TTV species were associated with graft rejection or viral infection.

The number of TTV species detected did not correlate with the risk of graft rejection or viral infection. Pearson’s correlation coefficients were r = 0.112 (*p* = 0.858) between the number of TTV species and graft rejection and r = −0.308 (*p* = 0.526) between the number of TTV species and viral infection.

### 3.4. Other Anelloviruses Detection

Other anelloviruses (such as TTMV and TTMDV) were detected in the plasma samples.

TTMV was simultaneously detected with TTV in a sample obtained 6 months post-transplantation. The same patient had only TTV in the pre-transplant sample.

TTMDV was simultaneously detected with TTV in two patients at months 6 and 12 post-transplantation.

None of the three patients with either TTMV or TTMDV developed viral infection or graft rejection (Table 3). The presence of these anelloviruses was only detected using the TTV R-GENE^®^ PCR.

## 4. Discussion

In the healthy human virome, one of the most frequent viruses is TTV, a member of the highly diverse *Anelloviridae* family. There are 22 TTV species (including more than 100 strains), divided into five genogroups (I, II, III, IV and V).

TTV is highly prevalent in the general population (90%) and can reach 100% in renal transplant patients. Studies performed in South America (specifically Brazil and Uruguay) in patients with HCV, HBV or HIV infection have reported that genogroup III species (TTV 13–24) were the most prevalent, followed by species from genogroup I (TTV 1–5) [24,25].

In renal transplant recipients from France and Italy, the most prevalent TTV species was TTV 3 (genogroup I). For French recipients, this was followed by former TTV 27 (now TTV 29, genogroup IV), TTV 24 and TTV 22 (both from genogroup III). In Italian recipients, TTV 13, TTV 15 and TTV 24 followed [23,34].

In our study, plasma samples (taken before and/or after renal transplantation) from a previously studied cohort [35] were selected to determine the TTV species diversity in this subpopulation and the variability over time in three of these patients. Genotyping was performed by whole-genome sequencing, using the RCA-NGS method and ORF1 phylogenetic analysis.

We were able to detect 15 TTV species, being TTV3 and TTV 24 the most prevalent.

In our study, high TTV species diversity was found. Specifically, up to 10 TTV species were detected in a single patient. In contrast, a single TTV species was detected in only three samples.

A high diversity of anelloviruses (mostly TTV) was previously described in blood and tissue samples taken from multiple sites within the same patient, including coinfections with closely related members of the *Anelloviridae* family (TTMV and TTMDV) [36,37]. In three samples from three different patients, TTMV or TTMDV were simultaneously detected along with TTV at different times post-transplantation.

TTV viral load, determined in plasma samples, has been associated with the immunosuppression state in renal transplant patients [23,35,38]. Therefore, TTV viral load can be used as a biomarker of immunosuppression in transplanted patients.

Given the abundance of TTV species described, a possible hypothesis was that a higher TTV viral load would indicate a higher number of TTV species present. In our study, no correlation between viral load and number of species was found. However, a significantly higher TTV species diversity was detected in the pre-transplant samples compared to post-transplant and in patients with grafts from a deceased donor compared to patients with a living donor.

This high diversity could be attributed to multiple factors, such as blood transfusions, the age of recipients, the earlier start of immunosuppression maintenance therapy in patients with a living donor or/and increased exposure to TTV infection during dialysis.

None of the patients in our cohort had received blood transfusions. Patients with a deceased donor started immunosuppression maintenance as late as 8 days post-transplantation; therefore, their immune system should have been able to control the TTV viral infection more efficiently. However, the observed higher TTV diversity could be attributed to the virus’ evasion mechanisms. Patients with a living donor initiated immunosuppression maintenance before transplantation, causing the initial lymphocyte depletion. This could explain the lower diversity, since there are fewer cells in which TTV can replicate.

Despite previous reports of TTV prevalence increasing with age and the possibility of higher exposure to different TTV species throughout life, no association between the number of TTV species and the age of the recipients was found in our study. Moreover, it was not possible to determine an association between the number of TTV species and the risk of graft rejection or viral infection.

For variability studies (evaluating the same patient at different time points), Kulifaj et al. described a high variability of TTV species when comparing pre- and post-transplantation samples [34]. In our study, all patients showed a variation in the number and type of TTV species detected using these experimental methodologies.

However, in this study, comparison between pre- and post-transplantation for the same individual was limited to three patients. Further studies, including a larger cohort with pre-and post-transplantation samples, are needed to better understand how TTV species change over time and their clinical relevance.

TTV replicates in mononuclear cells, mainly in lymphocytes [12,39]. Renal transplant immunity is largely diminished by both induction immunosuppression therapy (which causes a pronounced initial depletion) and immunosuppression maintenance throughout the post-transplant period. This could explain the observed variability of TTV species over time in the same host. Whether the detection of different TTV species is related to changes in the immune status remains to be clarified.

Since TTV is the most prevalent component in the healthy human virome and multiple species can be concomitantly detected within the same host, it is reasonable to consider that not all TTV species can equally affect or impact the human host. Therefore, it is important to further investigate which species are the most prevalent across the globe, their interaction with the host’s immune system and possible changes in the quantity and proportion of each TTV species over time in the same host.

The limitations of this study are the low number of samples genotyped and the single-center design. However, we were able to demonstrate that a combination of techniques like RCA, UAvI-PCR and NGS is a valuable tool for obtaining whole-genome TTV sequences. This combination was previously used by Spezia et al., 2023 [29] to successfully identify TTV species in patients with Kawasaki disease. These tools will contribute to a deeper understanding of TTV viral epidemiology due to the small number of studies currently available.

Research in this area is ongoing and has become increasingly established since TTV has been proposed as a biomarker of immune status. TTV determination may be an important factor in elucidating viral–host interactions in relation to immune responses.

## Figures and Tables

**Table 1:** Clinical and demographics characteristics of renal transplant patients (*n* = 27) with TTV sequencing.

Characteristics	Renal Transplant Patients (*n* = 27)
**Age, years old** [mean ± SD]	49.4 ± 13.9
**Gender (female)** [*n* (%)]	14 (51.8)
**Number of kidney transplantation** [*n* (%)]	
First	22 (81.5)
Second	4 (14.8)
Third	1 (3.7)
**Cause of end-stage renal disease** [*n* (%)]	
Diabetes mellitus	5 (18.5))
Glomerular disease	7 (25.9)
Hypertensive kidney disease	2 (7.4)
Polycystic kidney disease	2 (7.4)
Other specified disorders of the kidney and ureter	2 (7.4)
Unspecified chronic renal failure	9 (33.3)
**CMV serostatus** [*n* (%)]	
D+/R+	22 (81.5)
D−/R+	2 (7.4)
D+/R−	3 (11.1)
D−/R−	-
**Pre-transplant renal replacement therapy** [*n* (%)]	
Hemodialysis	23 (85.2)
**Time on dialysis, months** [median (IQR)]	30.6 (16.3–53.2)
**Donor age, years old** [mean ± SD]	43.5 ± 20.2
**Donor gender (male)** [*n* (%)]	17 (63.0)
**Type of donor** [*n* (%)]	
Deceased donor	20 (74.1)
Living donor	7 (25.9)
**Number of HLA mismatches** [median (IQR)]	5 (4–5)
**Induction therapy** [*n* (%)]	
Thymoglobulin	26 (96.3)
Basiliximab	1 (3.7)
**Initial maintenance immunosuppression scheme** [*n* (%)]	
Tacrolimus, mycophenolic acid and steroids	27 (100)

**SD**: standard deviation; **IQR**: interquartile range; **D:** donor **R:** recipient.

**Table 2:** TTV species prevalence in 30 plasma samples from 27 renal transplant recipients. Clinical and demographics characteristics of renal transplant patients (*n* = 27) with TTV sequencing.

TTV Species	*n* Samples	Prevalence (%)
TTV 3	19	63.3
TTV 24 (TTV 22 *, TTV 23 * and TTV 24)	16	53.3
TTV 9 (TTV 9, TTV 11 * and TTV 12 *)	11	46.7
TTV 13	13	43.3
TTV 18	13	43.3
TTV 5	11	36.7
TTV 1	9	30.0
TTV 15 (TTV 15 and TTV 16 *)	9	30.0
TTV 29 (TTV 27 *, TTV 28 * and TTV 29)	9	30.0
TTV 21	6	20.0
TTV 17	3	10.0
TTV 19	3	10.0
TTV 20	3	10.0
TTV 6	1	3.3
TTV 10	1	3.3

* Former classification.

**Table 3:** TTV viral load and TTV species in plasma samples from renal transplant patients (*n* = 27) using two PCR assays.

*n* Patients	ID Samples	Time after Transplantation (month)	TTV Viral Load (Log_10_ copies/mL)		*n* TTV Species	Anelloviruses		Viral Infection (Virus, Days after Transplantation)	Graft Rejection (Days after Transplantation)
			Home Brew PCR	R-GENE^®^ PCR		TTV Species	Other Anelloviruses		
1	**M 1_50945**	Pre Tx	5.4	5.0	6	3, 13, 15, 17, 18, 29	-	NO	NO
2	**M 2_48365**	Pre Tx	4.3	3.9	8	1, 9 (11/12), 13, 17, 18, 19, 21, 24	-	NO	NO
3	**M 3_10226**	Pre Tx	5.0	3.9	5	1, 3, 9 (11/12) *, 13, 24 (22/24) *	-	NO	NO
4	**M 4_50378**	Pre Tx	4.7	4.0	6	3, 9 (12), 13, 15 (16), 18, 24	-	NO	NO
5	**M 5_46007**	Pre Tx	4.4	4.0	4	13, 15 (16), 18, 24	-	NO	NO
6	**M 6_53410**	Pre Tx	4.2	2.9	4	3, 9 (12), 15 (16), 20	-	YES (CMV, +163)	YES (+38)
7	**M 7_48016**	Pre Tx	5.6	4.9	8	1, 3, 5, 9 (11/12) *, 10, 13, 19, 24	-	NO	NO
8	**M 8_10348**	Pre Tx	4.6	4.2	5	3, 13, 18, 24, 29	-	YES (BKPyV, +101)	NO
9	**M 9_49489**	Pre Tx	4.3	4.1	10	3, 5, 13, 15 (16), 17, 18, 20, 21, 24 (22,24), 29	-	NO	NO
10	**M 10_51173**	1	3.9	0.0	3	3, 9 (12), 13	-	NO	NO
11	**M 11_49748**	1	4.6	2.3	7	1, 5, 9 (12), 13, 15 (16), 24 (22), 29	-	NO	NO
12	**M 12_53806**	3	8.5	7.0	5	3, 5, 15, 18, 21	-	YES (CMV, +105)	NO
13	**M 13_49964**	3	8.2	7.8	4	1, 3, 18, 21	-	NO	YES (+220)
14	**M 14_51577**	3	8.2	7.7	7	3, 6, 9 (11), 18, 21, 24, 29 (28)	-	YES (BKPyV, +156)	YES (+36)
15	**M 15_50343**	3	8.5	7.7	2	15 (16), 29 (28/29) *	-	NO	NO
16	**M 16_47955**	3	8.5	6.7	2	3, 5	-	NO	NO
17	**M 17_** **46592**	3	8.2	7.2	7	3, 5, 9 (11), 13, 18, 24 (22), 29 (28)	-	YES (CMV, +221)	NO
18	**M 18_48748**	6	3.7	6.1	2	3, 5	TTMDV	NO	NO
19	**M 19_49833**	6	8.5	6.9	1	3	-	NO	NO
20	**M 20_49667**	6	7.3	5.5	7	1, 3, 9, 18, 21, 24, 29 (27)	-	NO	NO
21	**M 21_10232**	6	8.4	7.6	2	3, 24	-	NO	YES (+52)
22	**M 22_10175**	12	4.1	7.1	5	1, 5, 13, 18, 21	TTMDV	NO	NO
23	**M 23_11085**	12	6.9	5.0	2	20, 24 (22)	-	NO	YES (+86)
24	**M 24_10839**	12	8.6	7.2	2	5, 24	-	NO	NO
25	**M 25_46868**	Pre Tx	4.0	3.4	4	1, 3, 9 (12), 13	-	NO	NO
	**M 26_51364**	12	8.4	7.2	1	19	-		
26	**M 27_51795**	Pre Tx	3.6	4.3	5	1, 5, 15 (16), 18, 29	-	NO	NO
	**M 28_10274**	6	3.0	8.9	0		TTMV		
27	**M 29_46175**	Pre Tx	6.4	5.5	2	3, 5	-	YES (BKPyV, +307)	NO
	**M 30_49183**	9	8.1	7.2	1	21	-		

Pre Tx: pre-transplant sample. TTMV: Torque Teno Mini Virus; TTMDV: Torque Teno Midi Virus. (): TTV species assigned denomination in the previous taxonomical classification. (/) *: variants of TTV species named according to the previous taxonomical classification.

## Data Availability

The data presented in this study are available on request from the corresponding author (N.S.R., noeliasoledadreyes@gmail.com).

## Supplementary Materials

The following supporting information can be downloaded at https://www.mdpi.com/article/10.3390/v16030432/s1. Figure S1: Association between the number of TTV species and patient age; Figure S2: Association between the number of TTV species and time on dialysis; Table S1: Number of TTV species in plasma samples from renal transplant recipients (pre- and post-transplantation).
