# Supplementary material for: Torque Teno Virus (TTV) in Renal Transplant Recipients: Species Diversity and Variability
Source: Viruses. 2024 Mar 11;16(3):432. doi: 10.3390/v16030432 (PMC10974959; doi:10.3390/v16030432)

SUPPLEMENTARY MATERIAL

**Table S1**. Number of TTV species in plasma samples from renal transplant recipients (pre- and post-transplantation).

| **n Patients** | **ID Samples** | **Age** | **Type of donor** | | **Dialysis before transplantation** | | **Time after transplantation (month)** | | **n TTV species** | |  |
| --- | --- | --- | --- | --- | --- | --- | --- | --- | --- | --- | --- |
| 1 | **M 1_50945** | 34 | Deceased | | YES | | Pre Tx | | 6 | |  |
| 2 | **M 2_48365** | 36 | Deceased | | YES | | Pre Tx | | 8 | |  |
| 3 | **M 3_10226** | 40 | Deceased | | YES | | Pre Tx | | 5 | |  |
| 4 | **M 4_50378** | 40 | Deceased | | YES | | Pre Tx | | 6 | |  |
| 5 | **M 5_ 46007** | 44 | Living | | NO | | Pre Tx | | 4 | |  |
| 6 | **M 6_ 53410** | 55 | Deceased | | YES | | Pre Tx | | 4 | |  |
| 7 | **M 7_48016** | 57 | Deceased | | YES | | Pre Tx | | 8 | |  |
| 8 | **M 8_ 10348** | 62 | Living | | NO | | Pre Tx | | 5 | |  |
| 9 | **M 9_ 49489** | 68 | Deceased | | YES | | Pre Tx | | 10 | |  |
| 10 | **M 10_51173** | 61 | Living | | YES | | 1 | | 3 | |  |
| 11 | **M 11_49748** | 50 | Deceased | | YES | | 1 | | 7 | |  |
| 12 | **M 12_53806** | 28 | Deceased | | YES | | 3 | | 5 | |  |
| 13 | **M 13_49964** | 41 | Living | | YES | | 3 | | 4 | |  |
| 14 | **M 14_51577** | 62 | Deceased | | YES | | 3 | | 7 | |  |
| 15 | **M 15_50343** | 64 | Deceased | | YES | | 3 | | 2 | |  |
| 16 | **M 16_47955** | 68 | Deceased | | YES | | 3 | | 2 | |  |
| 17 | **M 17_46592** | 79 | Deceased | | YES | | 3 | | 7 | |  |
| 18 | **M 18_48748** | 39 | Deceased | | YES | | 6 | | 2 | |  |
| 19 | **M 19_49833** | 43 | Deceased | | YES | | 6 | | 1 | |  |
| 20 | **M 20_49667** | 49 | Deceased | | YES | | 6 | | 7 | |  |
| 21 | **M 21_10232** | 54 | Deceased | | NO | | 6 | | 2 | |  |
| 22 | **M 22_10175** | 25 | Deceased | | YES | | 12 | | 5 | |  |
| 23 | **M 23_11085** | 40 | Living | | NO | | 12 | | 2 | |  |
| 24 | **M 24_10839** | 45 | Deceased | | YES | | 12 | | 2 | |  |
| 25 | **M 25_46868** | 31 | Living | | YES | | Pre Tx | | 4 | |  |
|  | **M 26_51364** |  |  |  |  |  | 12 | | 1 | |  |
| 26 | **M 27_51795** | 50 | Deceased | | YES | | Pre Tx | | 5 | |  |
|  | **M 28_10274** |  |  |  |  |  | 6 | | 0 | |  |
| 27 | **M 29_ 46175** | 68 | Deceased | | YES | | Pre Tx | | 2 | |  |
|  | **M 30_49183** |  | |  | |  | | 9 | | 1 | |

Pre Tx: Pre-transplant sample

**Figure S1.** Association between the number of TTV species and patient’s age.


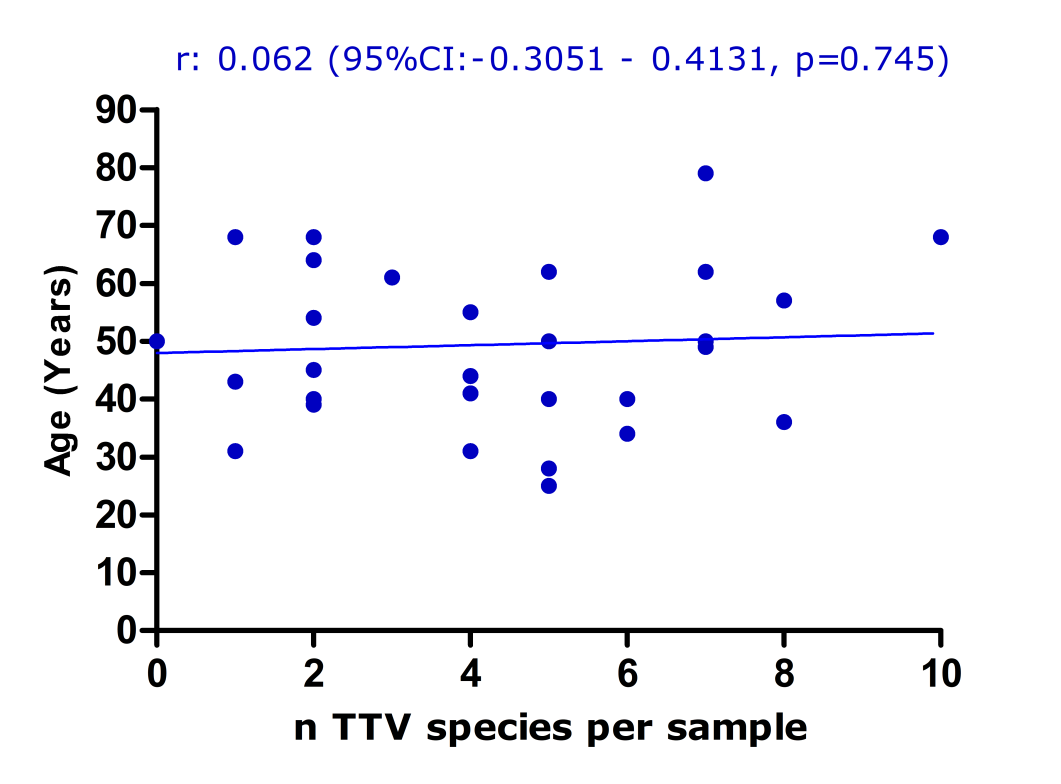


**Figure S2.** Association between the number of TTV species and time on dialysis


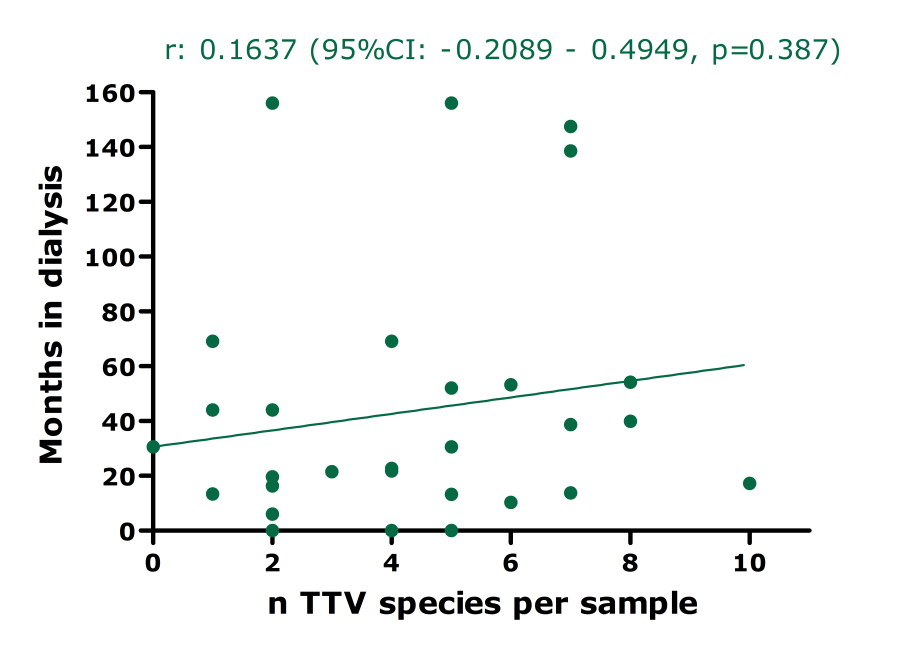

Supplement: Supplementary file 1 [file viruses-16-00432-s001.zip › viruses-2863420-supplementary.docx]
